# An Age-Friendly Living Environment as Seen by Chinese Older Adults: A “Photovoice” Study

**DOI:** 10.3390/ijerph13090913

**Published:** 2016-09-13

**Authors:** Aileen W.K. Chan, Helen Y.L. Chan, Ivy K.Y. Chan, Bonnie Y.L. Cheung, Diana T.F. Lee

**Affiliations:** 1 The Nethersole School of Nursing, The Chinese University of Hong Kong, New Territories, Hong Kong, China; aileenchan@cuhk.edu.hk (A.W.K.C.); tzefanlee@cuhk.edu.hk (D.T.F.L.); 2 Hong Kong Christian Service, Hong Kong, China; ivychan@hkcs.org (I.K.Y.C.); bonniecheung@hkcs.org (B.Y.L.C.)

**Keywords:** age-friendly cities, ageing in place, photovoice, senior housing

## Abstract

“Ageing in place” is a policy initiative strongly advocated by the World Health Organization to face the challenge of an ageing population. This pilot study used a “photovoice” approach, aiming to explore aspects of the housing environment considered by older people as important in facilitating ageing in place. It enabled participants to express their ideas through photographs. Each participant was asked to take photos that illustrated age-friendly features they considered crucial for supporting their lives in the community. A total of 44 older people participated in the pilot study, and 300 photos were collected. Participants were invited to describe the reasons for taking these photos by filling in a journal sheet. A semi-structured interview was then conducted with individual participants, who were asked to elaborate on the meaning of their photos. The analysis revealed three themes: (1) age-friendly housing design; (2) supportive neighborhood; and (3) connection to family and the community. These three themes are pillars of an age-friendly city, which are important to seniors to facilitate ageing in place.

## 1. Introduction

Demographic ageing is a global trend with significant implications for human development in the present century. The number of people aged 60 and over as a proportion of the global population will double from 11% in 2006 to 22% by 2050 [1]. People aged 65 and over accounted for more than 1.02 million or 14.2% in Hong Kong in 2013 [2], a proportion that will increase to 30.2%, or over 2.56 million, by 2041 [2]. Ageing in place is a policy which encourages older people to remain living in the locality they are familiar with for as long as they wish. It is an initiative strongly advocated by the World Health Organization (WHO) to face the challenge of an ageing population [3].

In Hong Kong, public housing is a main component of the overall housing stock, with nearly half the population now residing in some form of public housing [4]. It is important to understand the nature of desirable living conditions and the major elements conducive to a supportive environment from the perspective of people at retirement age. The World Health Organization (WHO) has introduced an age-friendly cities guide to engage cities in becoming more age-friendly [3]. The age-friendly city model initiated by the WHO reflects attempts to develop supportive urban communities for older citizens, which have been described as encourageing “active ageing by optimizing opportunities for health, participation and security in order to enhance quality of life as people age” [3]. The WHO guiding framework for an age-friendly city has eight themes covering a wide range of topics. This paper focuses on the housing environment: a key feature essential to personal mobility, safety and social participation, and well-being. The present pilot study, using a photovoice approach, aimed to explore elements in their housing environment considered by older adults to be important in encourageing ageing in place. This community-based participatory activity enabled older adults to express their ideas through photographs.

Photovoice is a process that uses pictures to identify themes and illustrate the needs of a given population. As part of this process, participants are asked to take photographs to document needs and concerns from their own viewpoints. They are then asked to write or verbalize individual narratives to complement their photographs [5]. A few studies have used photovoice with older adults. Baker and Wang (2006) used this methodology to gain a more personal understanding of older adults’ experience of chronic pain compared what could be achieved by the use of standardized assessment instruments [6]. Yankeelov et al. (2015) used photovoice to learn about older people living with diabetes [7], and LeClerc et al. (2002) used it to explore the struggles people encounter after hospital discharge [8]. Chaudhury et al. (2012) used photovoice as part of a larger effort to document the physical and social influences on physical exercise among older adults [9]. Minkler (2005) underscored the importance of this type of participatory action research with older adults [10]: findings have local relevance, their interpretation is inevitably more valid than can be achieved using other approaches, and they can be translated into culturally appropriate policies and programs. Photovoice is a creative approach that enables people to identify, define, and enhance their community according to their own specific concerns and priorities [5].

This photovoice study follows a project conducted by a research team in Australia, “My Place for Life: a comparison of models of care and housing for older people” [11]. Older participants in this study were asked to photograph various aspects of living spaces that were important to them and that enabled them to live in the community. Photovoice enabled the researchers to find out what worked well and what required improvement at the individual and community levels.

This study is the first photovoice project dealing with healthy older adults in Hong Kong. Participants narrated their experiences and expectations of their living environment, and how it affected their daily lives. The study aimed to explore elements considered by older people to be important in facilitating ageing in place. To address these aims, three research questions were formulated:

(1) What features of the housing environment are age-friendly?
(2) What features are experienced by older people as barriers?
(3) What improvement can be suggested for an age-friendly city?

## 2. Materials and Methods

### 2.1. Participants and Setting

The study included older adults who (i) were aged over 55; (ii) lived at their home; (iii) were physically and cognitively able to take digital photos; (iv) spoke Cantonese; and (v) owned a digital camera or a smartphone with a camera. We included subjects aged from 55 because 55–60 was the retirement age in Hong Kong, and would be the target population of age-friendly cities. To maximize variation, a purposive sample that included older people with variation in socio-demographic characteristics (e.g., age, gender, educational background and social class) was recruited from six district elderly community centers (DECCs) in Hong Kong. Ethical approval was obtained from the Survey and Behavioral Research Ethics Committee of the Chinese University of Hong Kong. Written consent, which included the research title, purpose of the research, and study procedures, was obtained from every eligible participant who agreed to participate. Each participant was given opportunities to ask questions, was free to refuse to answer any question, and might withdraw from the study at any time. Permission for the research team to use the photos that participants took for the study was obtained before the study began.

### 2.2. Procedure

The study procedure was in accordance with the Australian study [11]. After informed consent was obtained from the participants, they were asked to take photos of aspects of living in community-based housing or their own home that were important to them, using their own camera. They were encouraged to contact the researcher with any questions or concerns over the three-month study period. At the end of this time, the participants might have taken many photos for the study. The researcher contacted the participants and asked them to limit the number of photos to 10 that they believed were the most important. The researcher then visited each participant and downloaded the photos or the participant could send digital data to the researcher via email. The researcher printed the photos and sent a hard copy to the participant along with journal sheets and a letter with instructions. The participant was asked to complete a journal sheet for the photos. The journal consisted of 10 sheets of paper (one for each photo), on each sheet were printed the following three questions:

- What is in the photo? Please describe this photo.
- What does the photo mean to you (the photo taker)?
- Why did you take it?

The researcher then arranged an interview time with participant at their home (or other venue if the participant preferred). A one-hour, semi-structured interview was held, focusing on the 10 most important photos, and the participant was asked to elaborate on their meaning. With the participant’s consent, the interview was recorded digitally and transcribed verbatim. Both the journal sheet entries and the interview transcripts were coded and analyzed thematically.

### 2.3. Data Analysis

Qualitative content analysis was conducted to analyze the photos and captions. Content analysis is a method for categorizing the content of narrative communications in a systematic and objective fashion. Initially, the research team reviewed the qualitative comments and the photos independently, and then categorized the themes. The trustworthiness of the qualitative inquiry was ensured through investigator triangulation in the data analysis process. Triangulation included coding, condensation and abstraction [12]. Coding is classification or quantification of content, which can be explicit or implicit. Condensation involves a process that summarizes data. Abstraction is grouping data together under subheadings. To ensure the credibility, applicability, consistency, and neutrality of data, several measures were adopted. First, two researchers analyzed transcripts independently to identify significant statements and performed separate coding. Second, all interviews were conducted by the same moderator to ensure consistency. Third, an interview guide with three questions was used to maintain interview consistency. Lastly, member checking was performed to ensure the credibility of data interpretation. Member checking was the process performed at the end of interview through debriefings and discussions with the informant, to ensure that his/her answers had been properly understood. Any unclear dialogue was clarified.

For each of the topic areas, the analysis categories were age-friendly features, barriers, and suggestions for improvement. A final comparison between the theme descriptions and the tabulated data was performed to ensure that the analyses covered the reports from the participating subjects fully and accurately.

## 3. Results

A total of 44 older people participated in the study; their ages ranged from 63 to 82, with a mean of 70.6 ± 6.9. Of the 44 participants, 13 (30%) were men and 31 (70%) were women. More than half (57%) were married, and 25 (57%) had a primary education level or below. The majority (*n* = 37, 84%) were living in public housing. Demographic characteristics are summarized in Table 1.

The photos and captions were categorized into the following overarching themes. Three main themes were identified: (1) age-friendly housing design; (2) supportive neighborhood; and (3) connection to family and the community. The following section describes each theme, together with translated interview excerpts.

### 3.1. Age-Friendly Housing Design

The “age-friendly housing design” theme encompasses issues that relate to outdoor and interior housing design. Both public and private housing were perceived by the participants as structurally sound, and they appreciated having lift access to upper-level flats.

As to age-friendliness, two participants praised barrier-free facilities, such as the lobby entrance where there was sufficient space for a wheelchair-bound older person to move around:

The lobby entrance is of a height to allow the older person to use the ramp to go in and out, which is convenient. They may also use the railings.

(Informant 9)

The spacious lobby allows wheelchairs to move around.

(Informant 18)

Other participants praised features that support people with poor eyesight:

Good lighting of the corridor allows the older person with weak eyesight to see well, especially at night. I hope the lobby and corridor remain well lit.

(Informant 18)

The visually impaired, such as those who have lost their sight because of diabetes, may use the guided paths.

(Informants 12)

However, one participant criticized the design of a corridor:

The design of the corridor, with doors facing each other, causes embarrassment and inconvenience to users, for one door is opposite the other when they are open…. The Y-shaped building is a better design. It provides better spacing and privacy for residents as well as better daylight in the corridor.

(Informant 25)

Safety in the home environment was another concern:

There are sufficient fire safety facilities in the building, such as smoke doors and hoses. Centralized town gas is available, which is much safer than liquidized petroleum gas because it receives regular check-ups to minimize the chances of explosion and collapse.

(Informant 9)

However, some participants commented on safety issues with the flats’ interior design:

There are steps at the entrance of the toilet and kitchen, which is no good because older people need to raise their legs when they enter, which may increase the risk of a fall. I also hope that there are rails along the toilet wall to help me get up after finishing.

(Informant 13, 28 and 44)

Another participant preferred to have a user-friendly hanging rack for drying clothes:

There are only three bamboo sticks outside the window to dry the laundry. It is very inconvenient and dangerous when hanging clothes outside the window. It can be modified to a rack, which is safer.

(Informant 39)

Some participants felt insecure and feared living alone. One participant suggested that installation of a remotely-controlled door should be subsidized by the government for the disabled older people:

There is an option to install a remotely-controlled door and electric eye for the flats of the disabled or wheelchair-bound older people. When someone rings the doorbell, the bell sounds and a light flashes as well, which is convenient for those whose hearing or sight is impaired. The electric eye allows users to know who is outside the door. I think the government should subsidize the money to help older people for installing this device.

(Informant 30)

The theme of age-friendly design also encompasses issues that affect participants’ sense of comfort in their flats. Some participants were pleased to have a large balcony. Informants 9 and 35 valued having large balcony areas, which facilitated air circulation and provided pleasant views. They added that the “life-giving quality of a garden” is an important element that benefits their psychological well-being. Two informants used their balcony areas for flowers (Informants 9 and 30), and another one also valued having space to grow food:

Flowers are very lively. I plant flowers and tomatoes on my own. I am very happy to watch them grow.

(Informant 35)

Further, participants generally preferred a low-density housing estate, with pleasant views from the apartment, rather than seeing other high-rise buildings:

I see buildings from my window, which is difficult for me. The glass in their windows reflects light back to me, which affects my daily life. I hope that there is space between buildings, so that there is no shielding effect, and that I can live in a better environment.

(Informant 7)

### 3.2. Supportive Neighborhood

This theme covers facilities such as a wet market, supermarket, hair salon, post-box, pharmacy, and shopping mall. Older participants valued living close to services and facilities as an age-friendly feature. The seniors particularly preferred a wet market—a market that sells fresh meat and vegetables—to a supermarket, for the variety of choices and opportunity for face-to-face interaction with the vendors (Informants 2, 7, 9 and 38). Informants also commented:

Sometimes, the food at the supermarket is not as fresh.

(Informant 8)

I have been a customer of this stall for over 20 years. The owner is friendly and approachable. I have a good relationship with him.

(Informant 6)

Three residents mentioned the convenience of having access to a variety of shops in a mall, including: shops for clothes, furniture and daily essentials; restaurants; clinics and pharmacies; and hair salons. (Informants 2, 8 and 42)

### 3.3. Connection to Family and the Community

The “connection to family and the community” theme encompasses facilities that foster intergenerational interactions and events organized by community centers to encourage residents’ social participation. It is important for older people to retain links with family and community.

One commented happily on her participation in different festivals:

I am happy to see Buddhist parades and performances. I am also happy to spend time with my family during the mid-autumn festival.

(Informant 41)

Other than organizing outdoor activities during festivals, community centers also provide venues for different social and recreational programs. For instance, one subject commented:

Lok Wah Community Center has organized performances, as well as providing sports facilities for rent.

(Informant 4)

Another two participants said that they frequently sign up for interest groups, such as dancing and tai chi (Informants 7 and 37).

Aside from links with the community, older adults found their links with family particularly important, so that facilities that foster such links were highly valued. For instance, participants commented about nearby schools and playgrounds:

I hope the estate is not purely residential. It is important to have schools nearby.

(Informant 7)

I like that we have schools in the estate. It will be convenient for seniors to take grandchildren to school.

(Informant 11)

It is good to have the children’s playground, which allows the older people to take their grandchildren there and watch them playing in the playground.

(Informant 2)

## 4. Discussion

This study aimed to find out what aspects of the living space were important to older people. The findings of this study have provided insights into the lived experience of community-dwelling older adults. The overarching themes that emerged from the study were age-friendly housing design, having a supportive neighborhood, and connection to family and the community, which were regarded as important to age-friendliness. The community-dwelling older person relies heavily on the facilities provided by the housing estate, and it is important that these facilities are well maintained and age-friendly.

As mentioned earlier, Hong Kong public housing is a main component of the overall housing stock [4]. Residents in public housing do not own the flats, which must be returned to the Hong Kong Housing Authority after the person moves out or dies, if no one else is living there. However, residents are allowed to modify and personalize their flat at their own cost, after notifying the Hong Kong Housing Authority. The majority of older people (84%) in this study were living in a public housing flat.

With respect to age-friendliness, older people expressed the view that the outdoor environment was maintained better than the interior, where the lobby entrance was barrier-free, but the kitchens and toilets in the apartments were not. Participants had a good understanding of how their apartments could be modified to become age-friendly—for instance, having remotely-controlled doors, railings in the toilet, and barrier-free toilets and kitchens. However, these modifications are costly, and may not be affordable for low-income older people. Thus, subsidized programs should be available in order to allow older people to modify their flats so that they become more age-friendly.

Each public housing estate is self-contained, and located within walking distance of essential services and facilities, such as clinics, banks, pharmacies, wet markets or grocery shops, and with direct access to a hospital via bus or mini-bus. Neighborhood Elderly Centers (NECs) and DECCs are community centers for the older people that the participants rely heavily on to help them feel part of the local community. These community support services operate at the district level to enable the older people to remain in the community [13]. These centers provide services and organize activities for the older people, which promote their active participation and integration in the community. Services include community education, health education, volunteer development, and social and recreational activities [14]. The well-being of older adults may also be enhanced through promoting neighborhood social cohesion [15]. The findings of this study highlight the importance of the work done by NECs and DECCs.

Intergenerational interactions are also important for older people, who treasure time spent with their children and grandchildren. Thus, social activities and facilities that encourage such interactions are highly valued by the older people: for instance, schools nearby and a playground for their grandchildren.

This photovoice project was a participatory, empowering, co-learning process between academic researchers and community members. It was participatory in that each participant’s camera or smartphone acted as a research tool, putting the power to decide what was important in the hands of participants.

There is a need to develop an understanding of housing and care for older people to see what works well and where improvements may be made. Encourageing participants to document how their living environment affected their daily life, including experiences in their personal environment and community, was invaluable in gaining access to individual, social and community-level influences. Photovoice adds value to comprehensive needs assessment beyond what is realized in traditional investigator-driven survey research. This grassroots method documents participants’ daily lives in more depth and in a manner relevant to the immediate context. The information gathered and insights gained should prove useful in designing new housing for older people in the future.

In conclusion: The results of the study revealed that the design of age-friendly housing should consider not only the outdoor environment, but also the inside of the flat, which should be easily modifiable to become age-friendly. In this regard, subsidized programs should be considered for older people who need to modify their flats to become age-friendly. Also, there should be sufficient facilities to enable intergenerational interactions. Essential services and facilities should be located within walking distance of an age-friendly estate.

### Limitations

This pilot study has several limitations. Certain population groups were more likely to participate in the study—women, people with the capacity to be mobile, and those living in public housing. Moreover, only those who owned a camera or a smartphone with a camera were included. Thus, the voices of men and people who need assistance to be mobile were under-represented. The voices of poorer people who could not afford a camera or a smartphone, or those who did not know how to take photos, were not heard. Future studies should recruit even numbers of men and women, so that gender differences in their viewpoints could be explored. In addition, a digital camera should be provided to the participants in order to include the voices from older people from different social classes and a variety of backgrounds.

## 5. Conclusions

As ageing in place is a policy initiative strongly advocated by the WHO for facing the challenge of an ageing population, it is important to understand what constitutes a desirable living environment for older adults, and the major elements conducive to a supportive environment. In an attempt to develop an age-friendly city, older people's lives and experiences should be used as a starting point to identify desirable community services and support. The outdoor areas of housing estates are particularly age-friendly, but the interiors of the flats are not barrier-free yet. Access to essential services that maintain the community connections of older adults is an important age-friendly feature. Family ties remain an important aspect of the life of older adults, so that social activities and facilities that retain the links are highly valued. This pilot study provides preliminary insights into the future design of housing and living environments for older people in the community. Policy strategies for making a city more age-friendly require a clear assessment both of ways to engage older people in community redevelopment and of the barriers that might be encountered.

## Figures and Tables

**Table 1:** Demographic characteristics of participants.

Characteristics	*n* = 44
Age (years) ^†^	70.6 (6.9)
Sex	
Male	13 (29.5%)
Female	31 (70.5%)
Marital status	
Married	25 (56.8%)
Widowed	12 (27.3%)
Divorced	5 (11.4%)
Single	1 (2.3%)
Others (missing)	1 (2.3%)
No. of children ^†^	2.52 (1.29)
Educational level	
Primary or below	25 (56.8%)
High School	17 (38.7%)
Tertiary	2 (4.5%)
Living status	
Alone	15 (34.1%)
With spouse	15 (34.1%)
With family	7 (15.9%)
With children	6 (13.6%)
With other relatives	1 (2.3%)
Occupation status	
Retired	32 (72.7%)
Housewife	10 (22.7%)
Employed	1 (2.3%)
Unemployed	1 (2.3%)
Housing type	
Public	37 (84.1%)
Private	4 (9.1%)
Home ownership scheme	2 (4.5%)
Tenement	1 (2.3%)
Age of housing ^†^	21 (16.9)

Data marked with ^†^ is presented as mean (standard deviation), otherwise as frequency (%).

## Abbreviations

The following abbreviations are used in this manuscript:

DECCs	district elderly community centers
NECs	neighborhood elderly centers
NGO	non-governmental organization
WHO	World Health Organization
